# Acetate Revisited: A Key Biomolecule at the Nexus of Metabolism, Epigenetics and Oncogenesis—Part 1: Acetyl-CoA, Acetogenesis and Acyl-CoA Short-Chain Synthetases

**DOI:** 10.3389/fphys.2020.580167

**Published:** 2020-11-12

**Authors:** John R. Moffett, Narayanan Puthillathu, Ranjini Vengilote, Diane M. Jaworski, Aryan M. Namboodiri

**Affiliations:** ^1^Department of Anatomy, Physiology and Genetics, and Neuroscience Program, Uniformed Services University of the Health Sciences, Bethesda, MD, United States; ^2^Department of Neurological Sciences, University of Vermont College of Medicine, Burlington, VT, United States

**Keywords:** ACSS1, ACSS2, aspartoacylase, deacetylation, acetylation, NAT8L, N-acetylaspartate, transcription factor

## Abstract

Acetate is a major end product of bacterial fermentation of fiber in the gut. Acetate, whether derived from the diet or from fermentation in the colon, has been implicated in a range of health benefits. Acetate is also generated in and released from various tissues including the intestine and liver, and is generated within all cells by deacetylation reactions. To be utilized, all acetate, regardless of the source, must be converted to acetyl coenzyme A (acetyl-CoA), which is carried out by enzymes known as acyl-CoA short-chain synthetases. Acyl-CoA short-chain synthetase-2 (ACSS2) is present in the cytosol and nuclei of many cell types, whereas ACSS1 is mitochondrial, with greatest expression in heart, skeletal muscle, and brown adipose tissue. In addition to acting to redistribute carbon systemically like a ketone body, acetate is becoming recognized as a cellular regulatory molecule with diverse functions beyond the formation of acetyl-CoA for energy derivation and lipogenesis. Acetate acts, in part, as a metabolic sensor linking nutrient balance and cellular stress responses with gene transcription and the regulation of protein function. ACSS2 is an important task-switching component of this sensory system wherein nutrient deprivation, hypoxia and other stressors shift ACSS2 from a lipogenic role in the cytoplasm to a regulatory role in the cell nucleus. Protein acetylation is a critical post-translational modification involved in regulating cell behavior, and alterations in protein acetylation status have been linked to multiple disease states, including cancer. Improving our fundamental understanding of the “acetylome” and how acetate is generated and utilized at the subcellular level in different cell types will provide much needed insight into normal and neoplastic cellular metabolism and the epigenetic regulation of phenotypic expression under different physiological stressors. This article is Part 1 of 2 – for Part 2 see doi: 10.3389/fphys.2020.580171.

## Introduction

Acetate has generally been discounted or minimized in human fatty acid metabolism. This is most likely because humans have lower serum acetate levels than many other mammalian species including ruminants, which rely extensively on gut microbe-generated short-chain fatty acids for energy derivation. In ruminants, the bulk of plasma short-chain fatty acids including acetate, propionate and butyrate are derived from the fermentation of microbiota accessible carbohydrates (MACs) by gut microbiota. Humans do not generate the same level of short-chain fatty acids from MACs as ruminants, and further, some modern diets are often low in these non-digestible carbohydrates. Ruminants harbor microbiota that can break down and metabolize cellulose, whereas human-associated microbiota are limited to digesting MACs other than cellulose. These factors may have limited interest in the study of acetate in human biology for some time, but in the last decade renewed interest has been fostered by studies into the emerging regulatory roles played by this short-chain fatty acid. Ironically, it is the signaling and metabolic interplay between the gut and its associated microbiota and other organs including the heart and brain that have sparked renewed interest in the study of acetate in human health and disease, including cardiovascular disease and diabetes (Perry et al., 2016; Marques et al., 2017). This shift in focus from metabolic fuel to regulatory agent has been rapid as acetate is now associated with an array of biochemical functions beyond lipid synthesis. This two-part review will broadly cover both aspects of acetate metabolism; acetate as a short-chain fatty acid used in lipid synthesis and energy derivation, and on acetate as a key regulatory agent through which cells respond to changing physiological conditions, nutrient and oxygen deprivation and other signals and stressors. Part 1 will focus on systemic and local acetate production, and the enzymes that activate acetate to form acetyl-CoA. Part 2 of this review will focus on acetate in health, disease and oncogenesis. Because acetylation affects the structure and function of so many proteins, any discussion of acetate biology, or the “acetylome” as it has been referred to, will cover an extremely broad range of topics. As such, this review can only provide a somewhat selective overview of some of these relevant topics.

### Acetate, Coenzyme A and Acetyl-CoA

Acetate is the shortest chain fatty acid and represents the basic building block of all long-chain fatty acids and sterols through its association with coenzyme A (CoA). Like all fatty acids, acetate must be coupled to CoA in order to participate in enzymatic reactions. CoA is an essential carrier of activated acyl groups in organisms from Archaea to mammals. CoA is synthesized in a 5 step enzymatic process utilizing pantothenate (vitamin B_5_), cysteine and adenosine diphosphate (from ATP) [reviewed in Leonardi et al. (2005)]. CoA acts as a carrier for acyl groups ranging in size from acetate (2 carbons) to very long-chain fatty acids in excess of 22 carbon atoms (Watkins, 1997). Long-chain fatty acyl groups are transferred to CoA via acyltransferases, whereas acetate is transferred to CoA by enzymes traditionally known as acetyl-CoA synthetases.

**FIGURE 1:**
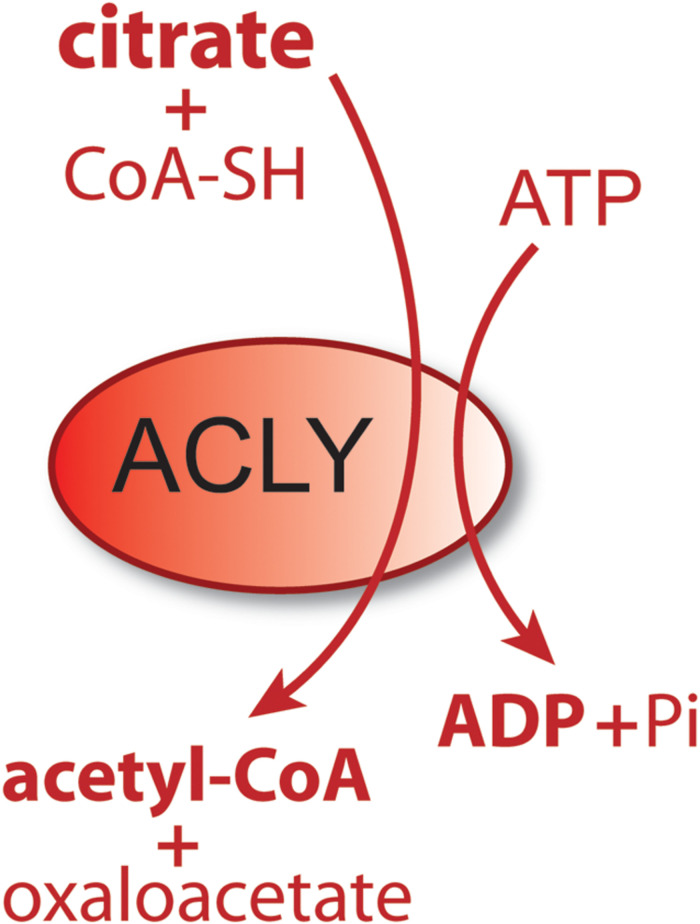
Schematic of the enzymatic reaction catalyzed by ATP-citrate lyase (ACLY). In the cytoplasm and nucleus of cells, citrate reacts with coenzyme A (CoA) and ATP to form acetyl-CoA plus oxaloacetate, ADP and phosphate. Because this enzymatic reaction is energy requiring and results in a shift in the ratio of ATP/ADP (ATP reduced, ADP increased), it functions as part of a metabolic energy sensing system that exerts control over central metabolism and the shift from fed to fasted status.

Acetyl-CoA is the universal carbon currency at the intersection of energy derivation, energy storage and energy utilization in all cells, and is thought to have arisen very early in evolution (Russell and Martin, 2004). In eukaryotes, acetyl-CoA is synthesized extensively from pyruvate in mitochondria, which links mitochondrial metabolism to a wide array of extra-mitochondrial cellular functions ranging from lipid synthesis to enzyme and gene regulation through protein acetylation. It has been proposed that acetyl-CoA acts like a second messenger, whereby shifts in its intracellular levels affect numerous metabolic pathways [reviewed in Pietrocola et al. (2015)]. For example, increasing the concentration of acetyl-CoA causes shifts in histone acetylation patterns, implicating acetyl-CoA in exerting influence over site-specific chromatin remodeling (Henry et al., 2015). Because acetyl-CoA is required for a multitude of cytoplasmic and nuclear acetylation reactions, intra-mitochondrial acetyl-CoA must be moved to the cytoplasm and then to the nucleus. Mitochondria do not express acetyl-CoA transporters such as those found in the endoplasmic reticulum (ER), so acetyl-CoA cannot be exported directly to the cytoplasm. Instead, citrate, produced from acetyl-CoA in mitochondria, can be used for energy derivation in the citric acid cycle, or as a means of exporting carbon from mitochondria to the cytoplasm. Citrate is produced from acetyl-CoA in the mitochondrial matrix by the action of citrate synthase and is transported to the cytoplasm via the mitochondrial citrate carrier (CIC). In the cytoplasm, the enzyme ATP-citrate lyase (ACLY) converts citrate back into acetyl-CoA for use in fatty acid and sterol synthesis or protein and metabolite acetylation reactions. ACLY is the primary enzyme involved in generating extra-mitochondrial acetyl-CoA in the cytoplasm and nucleus of all cells (Chypre et al., 2012). Knockout of the *ACLY* gene is embryonic lethal indicating that there aren’t sufficient salvage pathways to overcome the loss of this enzymatic action during fetal development (Beigneux et al., 2004). The enzymatic reaction catalyzed by ACLY is shown in Figure 1. The reactants include citrate, CoA and ATP and the products are acetyl-CoA, oxaloacetate, ADP and phosphate. ACLY activity is coordinated with glucose availability, which is accomplished through ACLY activation via phosphorylation by the serine/threonine kinase AKT (also known as protein kinase B). In adipose tissue, insulin activates AKT, which in turn phosphorylates ACLY on serine 454, leading to ACLY activation and increased acetyl-CoA formation from citrate (Berwick et al., 2002; Lee et al., 2014).

**FIGURE 2:**
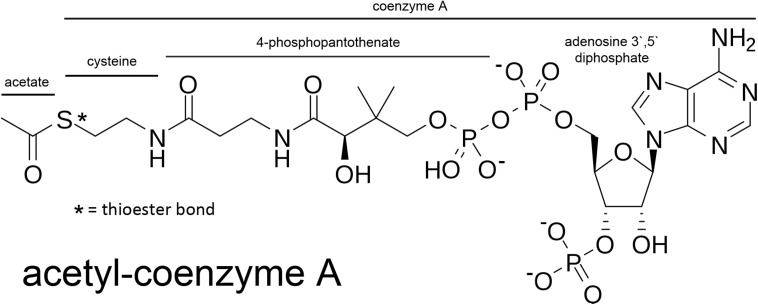
Acetyl-CoA and the segments derived from its basic constituents. Coenzyme A is comprised of adenosine 3′ 5′ diphosphate, 4-phosphopantothenate, and cysteine. The acetate group is bound to coenzyme A through a high-energy thioester bond (S; sulfur).

Acetyl-CoA is a relatively large, hydrophilic metabolite (MW ∼ 809; Figure 2). The thioester bond between the acetyl group and CoA is a high energy bond that is less stable than oxygen-based ester bonds (due to the larger size of the sulfur atom relative to an oxygen atom); thus making the transfer of the acetate group energetically favorable in enzymatic reactions. This facilitates acetate transfer to a vast array of acetylation targets. Due to its relatively large size and charge, acetyl-CoA does not cross membranes without the assistance of transporters. The *SLC33A1* gene encodes the ER integral membrane protein AT-1 (also known as ACATN1) which is the primary carrier of acetyl-CoA across the ER membrane to the luminal space (Jonas et al., 2010; Huppke et al., 2012; Pehar et al., 2012a; Hirabayashi et al., 2013). AT-1 is an evolutionarily conserved transmembrane protein widely expressed in various tissues. In contrast, acetyl-CoA transport from the cytoplasm to the cell nucleus occurs by simple diffusion through the nuclear pore complex. However, ACLY has been shown to be expressed in cell nuclei (Wellen et al., 2009) suggesting that acetyl-CoA might also be generated *de novo* within the cell nucleus. Nuclear acetyl-CoA is essential for histone acetylation reactions which are involved in chromatin remodeling and DNA repair (Campbell and Wellen, 2018; Sivanand et al., 2018). Nuclear generated acetyl-CoA is also essential for many other acetylation-based regulatory functions affecting gene transcription and protein activity. We will return to the topic of nuclear acetyl-CoA synthesis in Section “Acyl-CoA Short-Chain Synthetases and Acetate Utilization in Different Subcellular Compartments” below.

## Systemic vs. Local Acetate Production

**FIGURE 3:**
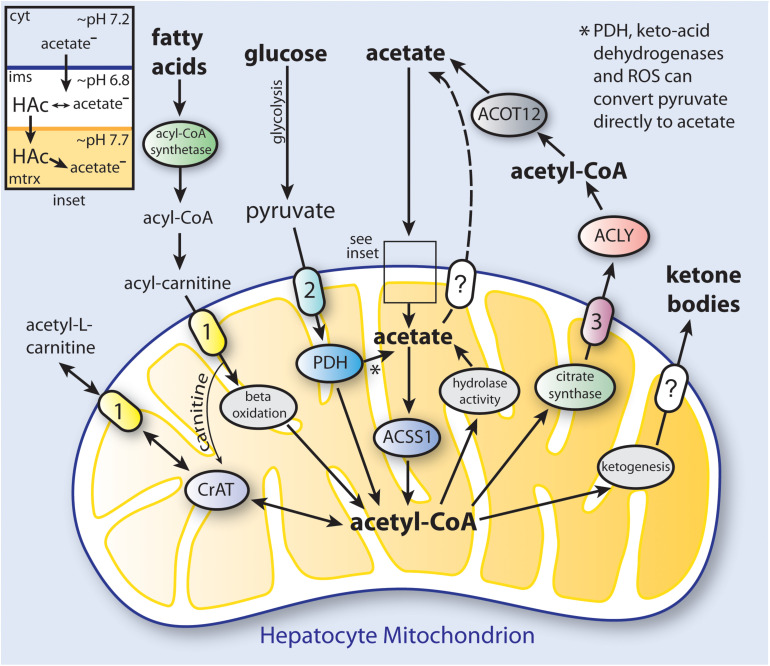
Simplified schematic of the distinct pathways to ketone body and acetate synthesis in hepatocyte mitochondria. In humans, hepatic production and release of acetate provides a significant amount of the acetate levels present in blood under ketogenic conditions. Triacylglycerols that are stored in hepatocyte lipid droplets, or that come to the liver from the circulation, are converted to fatty acids, which are then taken up by mitochondria and converted to acetyl-CoA through fatty acid β-oxidation. The acetyl-CoA produced in mitochondria can be utilized to synthesize ketone bodies (ketogenesis; see Table 2 for enzymes involved), or can be used to produce citrate which is exported to the cytoplasm. Cytoplasmic citrate is reconverted to acetyl-CoA by ATP-citrate lyase (ACLY), and the acetyl-CoA can then be hydrolyzed to free acetate by cytoplasmic acylthioesterase-12 (ACOT12). Acetate can also be transported out of mitochondria as acetyl-L-carnitine via the action of the reversible enzyme carnitine acetyltransferase (CrAT). When energy is required, acetyl-L-carnitine can be imported from the cytoplasm and reconverted to acetyl-CoA. Cytoplasmic acetate can gain access to the mitochondrial matrix when it is in the protonated form (HAc; see inset). PDH, keto-acid dehydrogenases and reactive oxygen species (ROS) can convert pyruvate to acetate (Liu et al., 2018). The mechanism of ketone body transport out of the mitochondrial matrix is currently unknown. It is also not known if acetate can be transported from the matrix by one of the mitochondrial carriers. The ketone bodies and acetate produced from acetyl-CoA in hepatocytes are then released to the circulation for use in other tissues. (1), carnitine-acylcarnitine translocase (CACT; SLC25A20); (2), mitochondrial pyruvate carrier (MPC1 and 2); (3), citrate carrier (CIC; SLC25A1); ACOT12, acyl-CoA thioesterase-12; CrAT, carnitine acetyltransferase; cyt, cytoplasm; ims, intermembrane space; mtrx, mitochondrial matrix; PDH, pyruvate dehydrogenase complex.

We propose that at least two types of mammalian acetate production, or acetogenesis, can be distinguished; systemic and local. Systemic acetogenesis occurs in tissues such as the intestine (resulting from the fermentation of fiber) and the liver during fasting, where much of the acetate is not used locally within the tissue of origin, but instead is released to the circulation for use in other tissues. Systemic acetate production and subsequent utilization are tissue specific. In contrast, local acetate production is the result of intracellular deacetylation reactions directed against acetylated metabolites and proteins. This acetate can be reconverted to acetyl-CoA and reused locally in all tissues. Systemic acetogenesis bears many similarities with ketogenesis, whereas local acetogenesis provides a means to recycle and utilize acetate derived from all deacetylation reactions. While the term acetogenesis is often used to refer to microbial generation of acetate via the Wood-Ljungdahl pathway (Ragsdale and Pierce, 2008), acetate production through a variety of biochemical pathways is also a hallmark of vertebrate metabolism (Figure 3).

**FIGURE 4:**
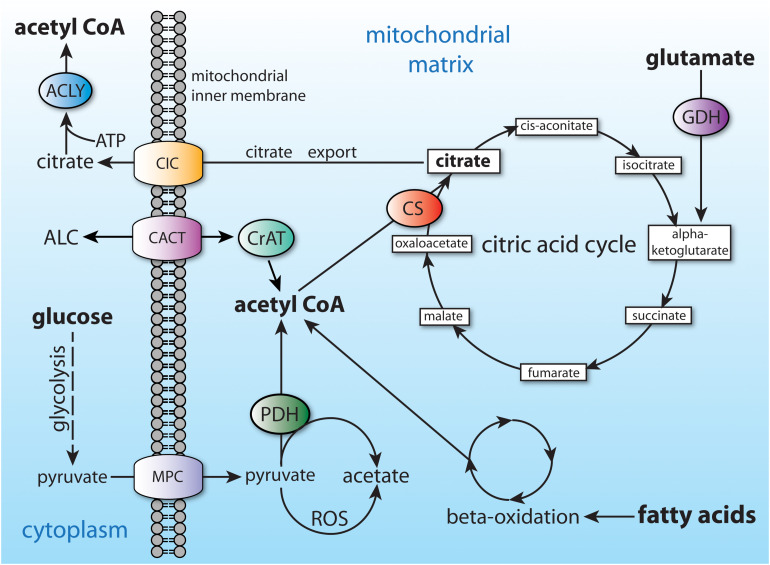
Central carbon metabolism and major sources of acetyl-CoA. Major inputs to acetyl-CoA synthesis include glucose (via glycolysis) and fatty acids (via beta-oxidation). Acetyl-CoA is also produced by entry of amino acids into the citric acid cycle (glutamate shown as an example). Acetyl-CoA cannot be transported from the mitochondrial matrix to the cytoplasm, and therefore is converted to citrate. The citrate can be utilized for energy derivation in the citric acid cycle, or it can be exported to the cytoplasm via the citrate carrier (CIC; *SLC25A1*). ACLY, ATP citrate lyase; ALC, acetyl-L-carnitine; CACT, carnitine-acylcarnitine translocase (SLC25A20); CIC, citrate carrier; CS, citrate synthase; CrAT, carnitine acetyltransferase; GDH, glutamate dehydrogenase; MPC, mitochondrial pyruvate carrier; PDH, pyruvate dehydrogenase.

In non-fasting humans, the primary source of acetyl-CoA in many cell types is glucose, which is converted to pyruvate through glycolysis. The pyruvate is then taken up by mitochondria and converted to acetyl-CoA by the action of the pyruvate dehydrogenase complex. Mitochondrial acetyl-CoA enters the citric acid cycle by combination with oxaloacetate to form citrate through the action of citrate synthase (CS; EC 2.3.3.1). Any citrate that is produced in excess of current cellular energetic requirements is exported to the cytoplasm (Figure 4). Transport of many metabolites into and out of mitochondria is made possible by members of the mitochondrial carrier family of transmembrane proteins, also known as the SCL25 protein family (reviewed in Palmieri, 2014; Palmieri and Monne, 2016). The SLC25 carrier family has 53 known members making it the largest solute transporter group in humans (Ruprecht and Kunji, 2020). Two dozen subfamilies of the SCL25 carriers are known to be expressed in eukaryotes, and these can be functionally subdivided into 4 general transporter groups for (1) amino acids, (2) nucleotides and dinucleotides, (3) carboxylates, and (4) other metabolites such as carnitine and acylcarnitine. Intra-mitochondrial citrate is exported via one of the SLC25 transporters known as the mitochondrial citrate carrier (CIC; gene *SLC25A1*), which exchanges intra-mitochondrial citrate or isocitrate for extra-mitochondrial malate.

However, there are no known transporters for acetate or ketone bodies in mitochondria (Puchalska and Crawford, 2017). Acetate is thought to gain entry to the mitochondrial matrix from the cytoplasm without the need of membrane transporters (Arduini and Zammit, 2017). Evidence indicates that acetate enters mitochondria by diffusion in the un-ionized form: acetic acid (Bernardi, 1999). There are substantial pH differences between the cell cytoplasm, the mitochondrial intermembrane space and the mitochondrial matrix (inset, Figure 3). Proton pumps in the inner mitochondrial membrane extrude H+, thus driving the pH of the matrix up to approximately 7.7, while reducing the pH of the intermembrane space to about 6.8. The pH of the matrix contributes to the proton motive force (Nicholls, 1974) and changes based on glucose availability (Akhmedov et al., 2010). The pH gradient drives the flux of metabolic substrates and has been observed to fluctuate rapidly using pH sensitive dyes (Santo-Domingo and Demaurex, 2012). The ratio of charged acetate (Ac^–^) to uncharged acetic acid (HAc) is pH dependent, and more acetic acid will be present in the intermembrane space (pH 6.8) than in the matrix (pH 7.7) thus creating a gradient that favors import into the matrix. However, the extent to which the acetate anion (Ac^–^) can be exported from the mitochondrial matrix via transporters is a poorly studied aspect of mitochondrial function. Fascinating results have been obtained using real time NMR of pure isolated mitochondria from a human colon cancer cell line (HCT116). Addition of 3 mM ^13^C_3_-labeled pyruvate resulted in acetate, acetyl-CoA and acetyl-phosphate formation (Xu et al., 2018). The formation of acetyl-phosphate was transient, suggesting that it was an intermediate that could be partly responsible for the steady increase in acetate production observed after pyruvate addition. Clearly, acetate is produced in mitochondria, but the extent to which, and the mechanism whereby it is transported to the cytoplasm, remain uncertain.

### Production of Systemic Acetate

Early research into the production and release of acetate from the livers of rats and sheep suggested that release occurred when the activity of the citric acid cycle was restricted, and that the function of acetate production was to redistribute oxidizable substrate throughout the body. The authors noted that this function was analogous to that served by ketone bodies (Knowles et al., 1974). In non-fasting humans, plasma acetate levels are relatively low, typically between 100 and 200 micromolar. In fasted humans, acetate accounts for approximately 5% of the free fatty acid turnover (Seufert et al., 1984). Interestingly, plasma acetate levels are higher in young adults (average 170 micromolar) than in adults aged 40 to 57 (average 130 micromolar). Further, 90% of the acetate turnover in both age populations led to CO_2_ production, indicating oxidation for energy derivation (Skutches et al., 1979). Acetate release and utilization are tissue specific. Under ketogenic conditions, the liver, kidneys, and intestine are net producers of free acetate, while the heart, skeletal muscle, and brown adipose tissue are major utilizers (Yamashita et al., 2001; Kondo et al., 2009; Sakakibara et al., 2009). In contrast to the more distributed production of free acetate, ketone bodies are produced predominantly in the liver, although some ketogenesis may also occur in the kidney (Nakatani et al., 1996; Nakatani et al., 1999). Early studies by Seufert and colleagues into the release of ketone bodies and acetate from isolated rat livers showed that in normal rats fed *ad libitum* the ratio of ketone bodies (acetoacetate plus β-hydroxybutyrate) to acetate ranged from 1.6 to 4 (Seufert et al., 1974). In the same study, hexanoate infusion through the portal vein (1 millimole per hour) increased the production of both acetate and ketone bodies several fold. Ketone body output increased from 95 to 850 nmoles/gm/min (over nine-fold increase). Acetate output increased from 29 to 140 nmoles/gm/min, which represents a 3.6-fold increase. Seufert and colleagues also found that the livers of alloxan-diabetic rats produced substantially more ketone bodies and acetate than did control rats before and during hexanoate infusion (Seufert et al., 1974). Additional studies in rats showed that isolated hepatocytes released substantial amounts of free acetate as a result of peroxisomal acetyl-CoA production when the hepatocytes were incubated with various oxidizable fatty acids (Leighton et al., 1989). Dietary addition of benzafibrate, to induce peroxisome proliferation, resulted in a six-fold increase in acetate release when lauric acid was provided as substrate. These findings indicate that in rats, peroxisomes are a significant site of free acetate production in the liver. However, the finding of acetate production and release by peroxisomes may not hold true for some other species, including humans.

Similar studies done in rats by Yamashita and coworkers using cannulation of the hepatic vein and portal vein showed that infusion of the short-chain (4 carbon) fatty acid butyrate resulted in more acetoacetate production than β-hydroxybutyrate (Yamashita et al., 2001). Infusion of longer chain fatty acids shifted the ratio in favor of β-hydroxybutyrate, wherein the ratio (β-hydroxybutyrate to acetoacetate) changed from 0.49 with butyrate infusion, to 4.45 with palmitate (C16) infusion. The studies by Yamashita et al. also provided ketone body and acetate production rates for whole perfused rat livers after 24 h of fasting. With butyrate infusion, the synthesis rates were 4.00 μmoles/min/liver for β-hydroxybutyrate, 8.20 μmoles/min/liver for acetoacetate, and 1.07 μmoles/min/liver for acetate. Upon palmitate infusion, the production rates were 10.03 μmoles/min/liver for β-hydroxybutyrate, 2.27 μmoles/min/liver for acetoacetate, and 2.63 μmoles/min/liver acetate. These results showed that with C16 fatty acid infusion the production rate for acetate exceeded that of acetoacetate, indicating the important role of acetate in redistributing oxidizable carbon sources coming from longer chain fatty acids.

Yamashita and coworkers further showed that livers co-produce acetate along with ketone bodies as products of β-oxidation, and that the acetate was not oxidized in the liver, but was oxidized in heart mitochondria (Yamashita et al., 2001). They showed that the acetate was produced from acetyl-CoA by the action of acetyl-CoA hydrolase, which was inhibited by CoA and activated by NADH. They concluded that acetate production is stimulated under ketogenic conditions by the low intramitochondrial level of free CoA, and the high level of NADH, both of which result from β-oxidation of fatty acids and the production of acetyl-CoA. It was estimated that acetate metabolism provides 8–10% of energy expenditure in dogs (Pouteau et al., 1998). Using radiolabeled acetate infusion, Pouteau and coworkers estimated that between 6 and 7% of basal energy expenditure was supplied by acetate in humans (Pouteau et al., 1996). In a pioneering study, Pouteau et al. used ^13^C-labeled short-chain fatty acids (less than 6 carbon atoms) in conjunction with gas chromatography/mass spectrometry to determine plasma concentrations and turnover rates for acetate, propionate and butyrate in human volunteers fasted overnight (Pouteau et al., 2001). This method yielded plasma concentrations of 181 + 28 μM acetate, 4.9 + 0.4 μM propionate, and 1.3 + 0.3 μM butyrate. Turnover rates were very low for propionate and butyrate, indicating that they are not oxidized extensively for energy derivation, whereas the turnover rate for acetate was 9.1 + 1.0 μmole/kg/min. The acetate turnover rate in rats was found to be approximately double the rate measured in humans. These findings again highlight not just species specific differences in acetate metabolism, but also that acetate is a significant redistributable carbon source in humans.

In further studies in humans, Pouteau and colleagues calculated rates of acetate production from gut fermentation vs. internal metabolism. The internal metabolic turnover rate was found to be a relatively constant 6 + 0.7 micromoles/kg/min, whereas that derived from gut fermentation after lactulose intake (lactulose is a synthetic non-digestible carbohydrate) reached a peak value of 3.2 + 0.4 micromoles/kg/min. They inferred that circulating acetate was therefore derived from two general sources; exogenous acetate from fermentation in the gut, and endogenous acetate arising from internal metabolism and turnover, with the endogenous source being the predominant one (Pouteau et al., 2003). As such, fermentation of MACs in the gut caused peaks in circulating acetate that were overlaid on a larger, more steady background rate. The importance of these findings lies in the fact that the bulk of circulating acetate can be derived from any food source that generates acetyl-CoA, not just from gut fermentation.

Systemic acetate production and utilization is an example of inter-organ metabolite exchange. Metabolite exchange between organ systems couples producer tissues with utilizer tissues. One of the major functions of the liver is as a producer of metabolites, vitamins, cofactors, amino acids, and glucose, which are released to the circulation for use in target tissues throughout the body. A classic example is the Cori cycle, in which lactate produced in muscles is transported to the liver where it is converted to pyruvate and then glucose, to be released back to the circulation (Katz and Tayek, 1998). Acetate and ketone bodies are also major players in this inter-organ exchange system. Recent studies by Jang and colleagues looked at metabolite exchange between organ systems by comparing metabolite concentrations in arterial blood vs. the venous blood draining from the various organs (Jang et al., 2019). Using anesthetized pigs, they identified 280 metabolites, with 91% of them showing statistically significant arteriovenous concentration differences for at least one organ system. Acetate was one of the metabolites identified, and in overnight fasted pigs, the major producers of acetate (higher acetate in venous blood) were the portal vein, colon, and head (brain). Organ systems that were net utilizers of acetate included the liver, spleen, kidney and leg (muscle).

The observation of acetate release from the head (carotid artery vs. jugular vein) is interesting and implies that the brain releases significant levels of acetate. However, the brain is also known to take up acetate efficiently from the bloodstream. In rats, the brain has been shown to take up and oxidize exogenously administered free acetate (Deelchand et al., 2009), and brain stimulation in awake rats increases acetate uptake and utilization (Cruz et al., 2005; Dienel and Cruz, 2006). Rowlands et al. (2017) found that acetate metabolism in the guinea pig brain includes both oxidation and incorporation into neurotransmitters such as glutamate and GABA. Acetate uptake and release has also been studied in humans. Using ^13^C-labeled acetate, Mittendorfer and colleagues showed that acetate was simultaneously produced by and released by the leg and splanchnic region, as well as in the whole body (Mittendorfer et al., 1998). Labeled carbon recovery in CO_2_ was almost identical in all three sampled regions, with values between 37 and 38% recovery. These findings highlight the dynamic nature of acetate redistribution, which is tied to shifting dietary and physiological conditions. For example, alcohol is converted to acetate in two enzymatic steps, and alcohol consumption increases acetate uptake in the human brain in a dose-dependent manner (Jiang et al., 2013; Volkow et al., 2013). This is in agreement with earlier findings that certain tissues take up and utilize acetate when blood levels are high, and tend to release acetate when blood levels are low (Mittendorfer et al., 1998). It will be very instructive for future studies to compare acetate redistribution between tissues in humans and other species, under different physiological and dietary conditions.

Whether a metabolite shows net uptake or net release from a particular tissue is a matter of several factors including plasma concentration, current synthesis and utilization rates, storage capacity and the localization and activity of suitable transporters within the tissue. For acetate, these factors can change in differing tissues depending on diet and nutrition, alcohol consumption, exercise, circadian cycle, injury or infection, fasting, etc. Clearly, sources and sinks for systemic acetate will shift as physiological conditions change over time.

#### Systemic Acetate Derived From Pre-existing Acetyl-CoA

Free acetate can be generated from a number of sources, with the major source being acetyl-CoA via the action of enzymes that have traditionally been referred to as acetyl-CoA hydrolases (EC 3.1.2.1). The major acetyl-CoA hydrolase is a cytoplasmic enzyme (Suematsu and Isohashi, 2006) involved in the generation and release of free acetate from the liver under ketogenic conditions. A newer nomenclature has been proposed for acyl-CoA hydrolase enzymes by Hunt and colleagues grouping them with acyl-CoA thioesterases, or ACOTs (Hunt et al., 2005). Under this newer designation system, the preferred nomenclature for the cytoplasmic form of acetyl-CoA hydrolase 1 is acyl-CoA thioesterase 12 (ACOT12). This form is responsible for the bulk of acetyl-CoA hydrolysis in cells.

The ACOTs liberate various chain length fatty acids from their CoA thioesters, and are critical enzymes involved in mobilizing fat reserves during starvation (Tillander et al., 2017). Suematsu and coworkers have cloned and sequenced the *ACOT12* gene from human liver and found that the gene encoded a 555 amino acid protein that was 81.4 and 78.7% identical to those of the mouse and rat, respectively (Suematsu et al., 2001, 2002; Suematsu and Isohashi, 2006). Western blotting and enzyme activity assays showed ACOT12 is preferentially localized in the cytosol in rats, but that a small proportion of the enzyme was also present in peroxisomes (Nakanishi et al., 1994). The enzyme contains a steroidogenic acute regulatory protein-related lipid transfer domain (START) at the C-terminus that downregulates activity via lipid binding (Horibata et al., 2013). Westin and colleagues showed that, in the mouse, ACOT12 is expressed at very high levels in the liver, kidney and proximal intestine epithelium (Westin et al., 2008). ACOT12 is primarily localized in the cytoplasm of hepatocytes indicating that the primary action is on cytoplasmic acetyl-CoA (Hunt et al., 2014). The β-oxidation of fatty acids acts to maintain acetyl-CoA supplies during fasting or starvation, and ACOT12 acts to help regulate cytosolic acetyl-CoA levels in hepatocytes. As such, ACOT12 generates free acetate for release to the circulation under ketogenic conditions, as is the case with ketone bodies. But the extent of the acetate exchange between different tissues, and between cells within a tissue, will vary between species. Numerous factors lead to substantial species-specific differences in acetate metabolism, including gut anatomy and microbiota, as well as the tissue distribution and subcellular localization of relevant enzymes and transport systems.

Additional routes to acetate formation have been elucidated. Studies by Liu and colleagues have shown that pyruvate dehydrogenase, the enzyme that converts pyruvate to acetyl-CoA, can also convert pyruvate directly to acetate (Liu et al., 2018). Further, they showed that reactive oxygen species, such as hydrogen peroxide, can convert pyruvate to acetate (see Figure 4). In an intriguing set of experiments, Liu et al. co-cultured acetate producing cells with ACLY deficient cells that require external acetate for viability. The acetate producing and acetate requiring cells were separated by a membrane that allowed small molecule diffusion. They showed that the acetate requiring cells were rescued by acetate released from the co-cultured acetate-producing cells, demonstrating intercellular acetate exchange directly.

Recently, fructose has been shown to be another important source of acetate production from gut microbiota. Zhao and colleagues have shown that when large amounts of fructose are consumed in mice that not all of it is taken up in the small intestine, but rather, some passes on to the colon where it is fermented into short-chain fatty acids, especially acetate (Zhao et al., 2020). Acetate levels in the portal vein doubled 60 to 90 min after fructose gavage, indicating that when dietary fructose intake is high, the increase in microbial generated acetate becomes substantial.

In sum, the major sources of systemic acetate are diet, gut fermentation and the hydrolysis of existing acetyl-CoA in certain tissues when fatty acid beta oxidation leads to acetyl-CoA generation in those tissues. In contrast to systemic acetate, locally-derived acetate is generated from an incredibly wide array of molecular sources. Local acetate production will be discussed in more detail in Sections “Local Acetate Production From Acetylated Proteins” and “Local Acetate Production From Acetylated Metabolites” below, and in part 2 of this review.

### Regulation of Acetogenesis and Ketogenesis

The degree to which systemic acetate production is metabolically important is species specific (Adams et al., 1997). This is due to differences in digestive system anatomy, unique microbiomes and distinct enzyme expression in the gut and liver. Hepatic acetate production and release are regulated in part by β-oxidation of fatty acids in hepatocyte mitochondria, which decreases CoA levels, and increases both acetyl-CoA and NADH, which act to increase acetyl-CoA hydrolysis by ACOT12 (Yamashita et al., 2001). Starvation has been shown to increase ACOT12 activity in the liver of rats (Matsunaga et al., 1985). Lipid signaling agents such as phosphatidic acid inhibit ACOT12 activity, and insulin acts to reduce ACOT12 mRNA and protein levels (Horibata et al., 2013). In contrast, ACOT12 activity in the liver is strongly upregulated by ATP, and is inhibited by ADP (Swarbrick et al., 2014). Therefore ACOT12 acts as a major source of hepatic acetyl-CoA hydrolysis and acetate production during lipid β-oxidation when ATP and acetyl-CoA levels are sufficient. Using hepatocellular carcinoma cells, Lu et al. showed that ACOT12 regulates both acetyl-CoA levels and histone acetylation (Lu et al., 2019). They found that as ACOT12 activity was reduced, acetyl-CoA levels and histone acetylation were increased, indicating the important roles of ACOT12 in regulating acetyl-CoA levels, as well as the generation of acetate.

#### Regulation Through PPAR

A major control point for ketogenesis is through the action of peroxisome proliferator-activated receptors (PPAR), which are transcription factors involved in the maintenance of energy homeostasis. There are three subtypes of PPAR (α, β/δ, and γ) with distinct but cooperative roles in regulating the metabolic transition to fasting (Dubois et al., 2017). All PPARs are involved in fat metabolism. PPARγ is expressed in white adipose tissue where it stimulates lipogenesis by up-regulating target genes such as fatty acid binding protein 4 and lipoprotein lipase (Patsouris et al., 2006). In contrast, PPARα activates genes associated with the mobilization of fat stores, oxidation of fatty acids, and generation of the end products of fatty acid catabolism including ketone bodies and acetate (Kersten et al., 1999). PPARα is expressed in tissues that are active in fatty acid oxidation including the liver, brown adipose tissue, heart and skeletal muscle (Kliewer et al., 1994; Lemberger et al., 1996). PPARα is activated by various ligands including fatty acids and eicosanoids (Forman et al., 1997; Chakravarthy et al., 2009). Once activated, PPARα forms a complex with transcription coactivators (Liang and Ward, 2006) and the transcription factor complex binds to peroxisome proliferative response elements to regulate gene transcription.

Low blood sugar and low insulin levels associated with fasting stimulate glucagon release from the pancreas. Glucagon is a 29 amino acid peptide hormone that acts to increase gluconeogenesis and mobilize fat stores during fasting. Studies by Longuet and colleagues have shown that glucagon is required for the metabolic response to fasting (Longuet et al., 2008). Using mice lacking the glucagon receptor (*Gcgr*−/− mice) they demonstrated that Gcgr initiated a genetic program that acts to increase fatty acid oxidation in a PPARα- and AMPK-dependent manner (AMPK = adenosine 5′-monophosphate-activated protein kinase). The role of PPARα and AMPK in the regulation of ketogenesis has been reviewed by Grabacka et al. (2016).

#### Regulation by Sirtuins

Additional control of acetogenesis, as well as ketogenesis, is mediated to a great extent by acetylation and deacetylation of mitochondrial and cytoplasmic enzymes. Protein acetyltransferases and protein deacetylases have profound regulatory effects on acetogenesis and ketogenesis during fasting. Relatively small changes are observed in mitochondrial protein expression levels in response to calorie restriction. However, mitochondrial protein acetylation profiles change dramatically in response to fasting or calorie restriction, demonstrating the critical role that protein acetylation plays in adjusting metabolism to caloric intake (Shimazu et al., 2010a,b). The focus of research in this area has centered on a class of enzymes known as Silent Information Regulator 2 (Sir2) enzymes, also known as sirtuins. Most sirtuins act as protein deacetylases.

There are seven known sirtuins (SIRT1 – 7) expressed in humans. Sirtuins are evolutionarily conserved NAD^+^-dependent protein deacetylases in eukaryotes that control aspects of metabolic flux and mitochondrial energy derivation. They also regulate links between caloric input, metabolism and gene expression [reviewed in Feldman et al. (2012)]. Because of their dependence on NAD^+^, these enzymes are in a key position to regulate energy metabolism (Yaku et al., 2018). Sirtuins play a pivotal role in regulating acetate metabolism through their interactions with two key enzymes known as acyl-CoA short-chain synthetases (ACSS). The ACSS enzymes are the only known mammalian enzymes that can convert free acetate into acetyl-CoA. ACSS1 and ACSS2 predominantly utilize acetate as substrate, whereas ACSS3 preferentially uses propionate (see Table 1). The activity of these enzymes is regulated, in part, by acetylation and deacetylation of specific lysine sites. Acetylation inactivates both ACSS1 and ACSS2, whereas deacetylation reactivates them. SIRT1 activates ACSS2 via deacetylation at lysine-661 in the cell cytoplasm and nucleus, whereas SIRT3 acts to activate ACSS1 in the mitochondrial matrix by deacetylation at lysine-635 (Hallows et al., 2006). In this manner, sirtuins regulate the conversion of free acetate to acetyl-CoA throughout the cell by activating the ACSS enzymes operating at the center of free acetate metabolism. The ACSS enzymes are discussed in detail in Section “Acyl-CoA Short-Chain Synthetases and Acetate Utilization in Different Subcellular Compartments” below, as well as in part 2 of this review.

**TABLE 1:** Estimated Km values for the three known ACSS enzymes toward the short chain fatty acids acetate, propionate, and butyrate.

	***Acetate***	***Propionate***	***Butyrate***
Acss1	0.6 mM	4.1 mM	>10 mM
Acss2	0.11 mM	3.4 mM	>10 mM
Acss3	5.4 mM	0.19 mM	3.7 mM

*Both Acss1 (mitochondrial matrix) and Acss2 (nucleus and cytoplasm) favor acetate as substrate, with low activity utilizing propionate and very low activity using butyrate. Acss3 (mitochondrial matrix) preferentially utilizes propionate as substrate. Data are from Fujino et al. (2001) (Acss1 and 2) and from Yoshimura et al. (2016) (Acss3). Somewhat different values were reported for Acss2 by Patel and Walt (1987): acetate = 0.05 mM, propionate > 10 mM, butyrate > 10 mM.*

SIRT3 activity is also an important regulator of ketone body metabolism. SIRT3 is localized in the mitochondrial matrix (Cooper and Spelbrink, 2008) and because of its control over acetyl-CoA formation from acetate in this compartment (via ACSS1) it is involved in metabolic fuel switching and ketone body utilization (Dittenhafer-Reed et al., 2015). Using quantitative acetylated protein measurements in five tissues, Dittenhafer-Reed and colleagues showed that fasted *SIRT3* knockout mice exhibit hyper-acetylated mitochondrial proteins relative to fasted wild type mice. The altered protein acetylation patterns were tissue specific, with fuel producing tissues such as liver and kidney showing a different acetylome response to *SIRT3* knockout and fasting than fuel utilizing tissues such as brain, skeletal muscle, and heart. In wild type mice, the liver and kidney expressed the highest protein levels of SIRT3, whereas brain, muscle and heart had lower expression levels. There was a positive correlation between SIRT3 wild type expression levels and the total number of mitochondrial protein acetylation sites in each tissue in fasted *SIRT3* knockout mice. Also, tissues that generally have lower SIRT3 expression (brain, muscle, and heart) exhibited larger fold changes in the acetylation status of mitochondrial proteins than tissues with high SIRT3 expression (liver and kidney). These findings indicate that SIRT3 reduces the protein acetylation load in mitochondria. Further, these investigators showed that in the brains of fasted *SIRT3* knockout mice less acetyl-CoA was formed from the ketone body acetoacetate indicating impaired ketone body metabolism (Dittenhafer-Reed et al., 2015). The authors note that two key enzymes involved in acetyl-CoA production from ketone bodies are likely targets for SIRT3 regulation through deacetylation. The first, succinyl-CoA:3-ketoacid-CoA transferase (OXCT) transfers CoA from succinyl-CoA to acetoacetate, forming acetoacetyl-CoA. The second enzyme, acetyl-CoA acetyltransferase 1 (ACAT1), then converts acetoacetyl-CoA into two molecules of acetyl-CoA. Both of these enzymes were found to be hyper-acetylated in the brains of *SIRT3* knockout animals indicating that SIRT3 facilitates ketone body utilization in target tissues such as the brain. Dittenhafer-Reed and colleagues conclude that SIRT3 mediates metabolic coupling between fuel producing and fuel utilizing tissues through deacetylation of key mitochondrial enzymes associated with ketone body utilization.

## Acyl-CoA Short-Chain Synthetases and Acetate Utilization in Different Subcellular Compartments

**FIGURE 5:**
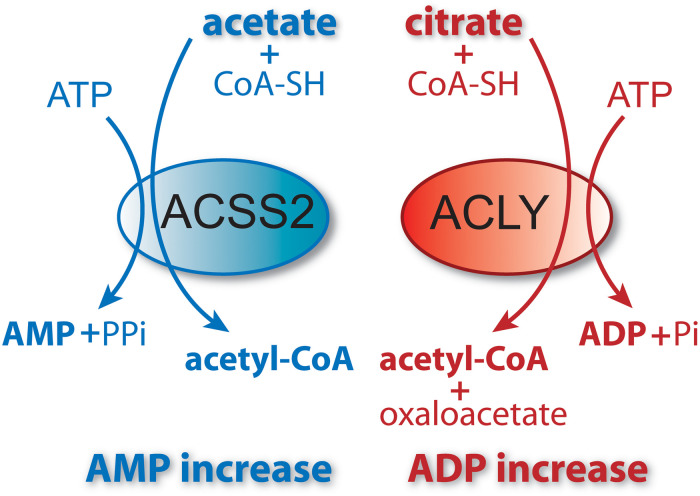
ACSS2 and ACLY act as parallel, but functionally distinct, pathways to cytoplasmic and nuclear acetyl-CoA formation. Citrate and acetate are both exported from mitochondria. ACLY uses mitochondrially synthesized citrate as substrate, whereas ACSS2 uses acetate from all sources. Both reactions are energy requiring and utilize ATP as cosubstrate. The end products of the ACLY reaction are acetyl-CoA, oxaloacetate, ADP and phosphate. The end products of the ACSS2 reaction are acetyl-CoA, AMP and pyrophosphate. As such, cellular and compartmental ADP/AMP ratios may depend, in part, on which of the two reactions predominates. Importantly, this has functional implications for metabolic regulation via enzymes such as AMP-kinase (AMPK). ACSS1 performs the same enzymatic reaction as ACSS2 but acts within mitochondria to generate acetyl-CoA for oxidation to CO_2_.

As is the case with all free fatty acids, unbound acetate can’t be further metabolized until it is coupled to CoA through a thioester bond. This reaction is catalyzed by ACSS enzymes, which expend ATP to generate the high energy thioester bond between acetate and CoA (Figure 5). Three known isoforms of acyl-CoA short-chain synthetases are expressed in humans encoded by the genes *ACSS1*, *ACSS2*, and *ACSS3* (Watkins et al., 2007). The enzymes are classified as acyl-CoA synthetase, short-chain family members 1 through 3 (EC 6.2.1.1). When the genes were designated, the names were not correlated with the known enzymes, resulting in a mismatch. The enzymes had been known as acetyl-CoA synthetases 1 and 2 (AceCS1 and 2) (Fujino et al., 2001). The nuclear-cytoplasmic form of the enzyme, known by the acronyms AceCS1 or ACS1, is encoded by the gene *ACSS2*, whereas the mitochondrial matrix form of the enzyme previously designated AceCS2 is encoded by the gene *ACSS1*. The remaining isoform of the enzyme, which is encoded by the gene *ACSS3*, has been localized to the mitochondrial matrix but this isoform preferentially utilizes propionate rather than acetate as its substrate (Yoshimura et al., 2016). Throughout the remainder of this review we will refer to the ACSS enzymes by their gene designations (non-italicized for proteins, italicized for genes).

The three known ACSS enzymes preferentially use either acetate or propionate as their primary substrates (Table 1). The two well characterized isoforms of the enzyme that use acetate as their major substrate, ACSS1 and ACSS2, are differentially expressed both in terms of their tissue distribution and subcellular localization. Early fractionation studies to determine the subcellular localization of total ACSS activity (designated at the time as acetyl-CoA synthetase activity) in rabbit tissues showed that activity was predominant in the cytoplasmic fraction of liver and the mitochondrial fraction of heart, whereas activity values were similar in the two fractions in kidney (Woodnutt and Parker, 1986). Because the Km values for acetate in both fractions were similar, these earlier investigators assumed that a single enzyme was responsible for activity in both the cytoplasm and mitochondria. It was not until later that it was determined that the isoforms in the heart and liver were distinct and were present in mitochondria in the heart and cytoplasm in the liver (Luong et al., 2000; Fujino et al., 2001). Their functional distinctions and intracellular localization patterns, wherein ACSS2 is associated with lipid synthesis and protein acetylation reactions and ACSS1 is associated with acetate oxidation, allow these enzymes to act in concert to store, mobilize, utilize, and recycle acetate throughout the cell.

### Acyl-CoA Short-Chain Synthetase Family Member 1

ACSS1 is a mitochondrial matrix enzyme that is strongly expressed in heart, skeletal muscle and brown adipose tissue. This isoform primarily generates acetyl-CoA that will enter the citric acid cycle for energy derivation via oxidation to CO_2_ (Fujino et al., 2001).

In brown adipose tissue, ACSS1 is involved in non-shivering thermogenesis under ketogenic conditions. In fasting *ACSS1*-deficient mice, there was a 50% reduction in skeletal muscle ATP, and the mice were hypothermic when fasted or fed a low carbohydrate diet (Sakakibara et al., 2009). In addition to demonstrating the link between ACSS1 and thermogenesis under ketogenic conditions, these findings also show that acetate is a vital energy-deriving metabolite when carbohydrate availability is low, as is the case with ketone bodies. During fasting, ACSS1 expression is increased by the transcription factors Kruppel-like factor 15 (KLF15) and trans-acting transcription factor 1 (SP1) (Yamamoto et al., 2004). ACSS1 expression in other tissues is relatively low when compared with muscle, heart and brown adipose tissue (Fujino et al., 2001). Enzyme assays of total acetyl-CoA synthetase activity, including activity from both ACSS2 and ACSS1, done on subcellular fractions from brain indicated that activity is lowest in the mitochondrial fraction (Waniewski and Martin, 2004), suggesting that the brain has a comparatively low capacity to utilize free acetate for energy derivation. However, it is possible that ACSS1 expression in the brain would be increased by fasting as was reported for brown adipose tissue, which showed a four-fold increase in *ACSS1* mRNA in fasted wild type mice (Sakakibara et al., 2009). Using immunohistochemistry, ACSS1 expression in the rat brain has been confirmed to be relatively low in normally fed animals and tentatively associated with astrocytes, including astrocyte processes and end feet that are in contact with endothelial cells of the vasculature and other cell types including oligodendrocytes (Moffett et al., 2013). This would place astrocytes in a position to take up blood-borne acetate, or acetate released from other cell types, such as oligodendrocytes, for use in the citric acid cycle. It is well documented that free acetate applied to the brain is preferentially taken up and metabolized by astrocytes for energy derivation (Cruz et al., 2005; Dienel and Cruz, 2006). The preferential uptake of acetate by astrocytes is mediated by a monocarboxylate transporter, and the accumulated acetate is rapidly converted to CO_2_ (Waniewski and Martin, 1998). This finding is in agreement with the preferential expression of ACSS1 in astrocytes. Acetate transport in the brain is mediated by several monocarboxylate transporters (MCT) including MCT1, MCT2, MCT4, and a sodium-dependent monocarboxylate transporter (SMCT1) (Rae et al., 2012).

### Acyl-CoA Short-Chain Synthetase Family Member 2

**FIGURE 6:**
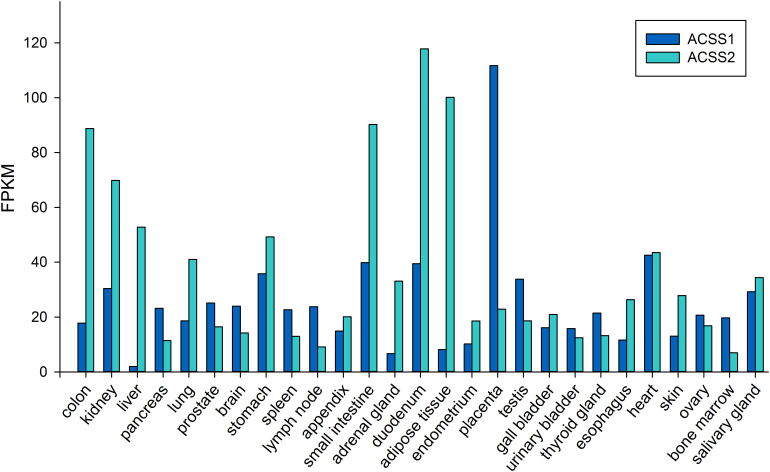
Expression levels of *ACSS1* and *ACSS2* from human tissues (expressed in FPKM; fragments per kilobase of exon model per million mapped reads). Data taken from supplemental online material in Fagerberg et al. (2014).

ACSS2 is a nuclear-cytoplasmic enzyme (Wellen et al., 2009; Ariyannur et al., 2010a). Early studies showed *ACSS2* expression to be high in the liver, kidney and heart, with moderate expression in brain and testes (Luong et al., 2000). To date, no systematic study has been undertaken to determine the distribution of ACSS2 in various tissues in the body. This is also true for the localization of ACSS1. The NCBI BioSample database^1^ shows ACSS1 and ACSS2 to be expressed in all of the human tissues that were examined. Higher expression levels for ACSS2 were found in muscle, kidney, small and large intestine and adipose tissue. Low to moderate expression levels were found in lung, liver and brain. The lowest expression levels for ACSS2 were observed in compartments of the immune system including bone marrow and lymph nodes. Using the data from genome-wide expression studies (Fagerberg et al., 2014) the expression of *ACSS1* and *ACSS2* in 27 human tissues is shown in Figure 6. Fagerberg et al. classified both enzymes as having generally low expression in all tissues. Because of its importance in utilizing intracellular acetate, we predict ACSS2 expression will be observed in virtually all cell types under different physiological conditions. Further, because ACSS2 is responsive to myriad physiological transitions, ranging from nutrient deprivation to injury and immune activation, we predict that ACSS2 expression in all cell types will be highly correlated with the degree of physiological stress. ACSS2 has multiple splice variants and isoforms, as well as post-translational modifications, and will no doubt turn out to be expressed differentially in tissues under various physiological contexts. Based on our own results with ACSS2 antibodies in different tissues, we expect that the different isoforms will not be recognized to the same extent by existing ACSS2 antibodies, and this may lead researchers to underestimate ACSS2 expression by immunohistochemistry.

ACSS2 acts as a parallel, but distinct pathway to ACLY (Figure 5) for the formation of cytoplasmic and nuclear acetyl-CoA (Wellen et al., 2009; Moffett et al., 2011, 2013; Zhao et al., 2016). In this way, ACSS2 provides for the recycling of acetate derived from deacetylation reactions in the nucleus, for example histone deacetylase (HDAC) reactions (Ariyannur et al., 2010a; Bulusu et al., 2017). This could provide efficient, local regeneration of acetyl-CoA in the nucleus by reducing reliance on acetyl-CoA derived from mitochondrial metabolism.

ACSS2 has long been associated with lipid synthesis (Howard et al., 1974). ACSS2 expression is induced by sterol regulatory element-binding proteins (SREBPs), transcription factors that regulate cholesterol and unsaturated fatty acid synthesis (Luong et al., 2000; Sone et al., 2002). When cholesterol levels in cells drop below a certain threshold, SREBPs are acted upon by proteases that generate active protein fragments that translocate to the nucleus to bind to sterol regulatory elements (SREs) and increase the expression of genes associated with cholesterol and fatty acid synthesis. *ACSS2* is one of the lipogenic genes in the liver that is upregulated by low cholesterol and fatty acid levels, and suppressed by high levels. Ikeda and colleagues reported that the *ACSS2* gene promoter has eight SRE motifs and that SREBPs act in conjunction with the transcription factor Sp1 to increase ACSS2 expression in response to lowered sterol and fatty acid levels (Ikeda et al., 2001). In many tissues, with the mammary gland of goats being one example, SREBP1 increases the expression of ACSS2 leading to increased lipid synthesis (Xu et al., 2017). Additional evidence for a role in lipid metabolism comes from studies on *ACSS2* knockout mice. *ACSS2* deletion does not result in obvious phenotypic differences and the knockout mice are fertile and produce normal litters (Huang et al., 2018). However, when the knockout mice were fed high-fat diets, they deposited less fat in adipose tissue and liver than wild type mice of the same strain, highlighting the role of ACSS2 in lipid synthesis. However, Huang and colleagues note that the role of ACSS2 in lipid synthesis appears more regulatory wherein it acts more like a transcription factor than a metabolic enzyme (Huang et al., 2018). They demonstrated that ACSS2 reprograms gene expression in multiple tissues to coordinate the physiological adaptation of animals to the fed or fasted state. This brings up an important caveat concerning the role of ACSS2 as a lipogenic enzyme. Most of these studies were done in liver, adipose tissue or on cells in culture, but the roles that ACSS2 plays in other tissues may be distinct from lipid metabolism. This topic will be discussed in more detail later in the review.

Compatible with its role in lipid synthesis, ACSS2 has also been implicated in regulating autophagy in a nutrient sensitive manner through acetyl-CoA formation and subsequent acetylation of proteins (Schroeder et al., 2014). Acetyl-CoA depletion induces autophagy whereas acetyl-CoA excess inhibits autophagy (Eisenberg et al., 2014). Marino and colleagues found that nutrient deprivation leads to substantial reductions in cytosolic acetyl-CoA levels in certain tissues, which in turn induces autophagy. They found that depletion of cytosolic, but not mitochondrial or nuclear acetyl-CoA, led to the induction of autophagy and the acetyltransferase EP300 was required for this action (Marino et al., 2014). In general, high concentrations of acetyl-CoA favor protein acetylation by acetyltransferases resulting in globally increased protein acetylation. Hyperacetylation of proteins subsequently inhibits autophagy by rapid and direct post-translational inactivation of proteins engaged in the autophagic response, by modulation of nutrient-sensing kinase pathways, and on a longer time scale through the epigenetic regulation of autophagy-related gene expression (Schroeder et al., 2014). Because ACSS2 contributes to the acetyl-CoA pool and is responsive to acetate levels, the intracellular level of acetate translates to increased ACSS2-mediated conversion to acetyl-CoA, which in turn drives protein acetylation reactions via acetyltransferases. Indeed, ACSS2 inhibition in cultured cells resulted in reduced acetyl-CoA levels, reduced cytoplasmic protein acetylation and increased autophagy (Marino et al., 2014). In this way, protein acetylation can be seen as a key sensor of nutrient status, linked to autophagy through protein acetylation levels, and ACSS2 supplies some of the acetyl-CoA required for these protein acetylation reactions.

**FIGURE 7:**
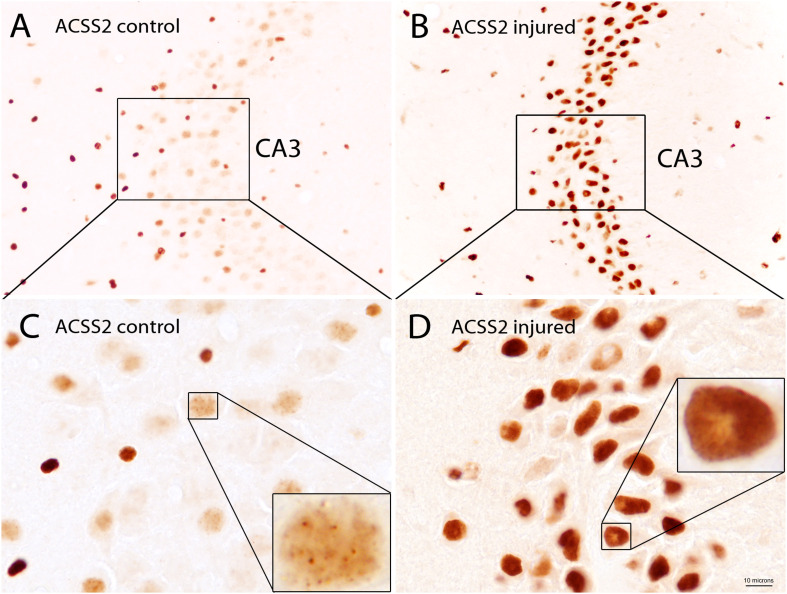
ACSS2 immunohistochemistry in the CA3 region of the hippocampus in a control rat as compared with a rat 3 days after unilateral controlled cortical impact injury (CCI). ACSS2 in the adult rat brain is expressed predominantly in cell nuclei, including those of neurons, astrocytes and oligodendrocytes. Staining in cell nuclei is highly variable between cells and non-uniform throughout the nucleus. Nuclear staining in all cell types is substantially increased throughout the brain after CCI injury. For example, in the principal neurons of the CA3 region of the hippocampus ACSS2 expression is substantially increased after brain injury: **(A)** Lower magnification image of the CA3 region of hippocampus from a control (uninjured) rat. **(B)** Lower magnification image from the CA3 region of hippocampus from a rat 3 days after CCI, on the side contralateral to the cortical injury. Images in **(C,D)** are higher magnification images of **(A,B)**, respectively. Images were taken of archival slides from Ariyannur et al. (2010a). Insets in **(C,D)** are enlargements of nuclei from principle CA3 neurons showing punctate ACSS2 staining associated with chromatin. These localized regions of increased staining in cell nuclei may represent accumulation of ACSS2 at promoter regions of activated genes. ACSS2 antibody concentration = 1:10,000. Bar = 10 microns in **(C,D)**; 30 microns in **(A,B)** and 2.5 microns in the insets in **(C,D)**. Details given in Ariyannur et al. (2010a).

The subcellular localization of ACSS2 is both cytoplasmic and nuclear in yeast (Falcon et al., 2010) and mammalian cells (Wellen et al., 2009) indicating the evolutionary conservation of function in both compartments. While detailed localization studies for the enzyme in various tissues throughout the body are lacking, ACSS2 expression has been examined in detail in the rat brain. In the adult rat brain, the predominant localization of ACSS2 is nuclear, rather than cytoplasmic (Ariyannur et al., 2010a). ACSS2 was expressed at substantially higher levels during postnatal brain development than in the adult brain. Further, the expression of ACSS2 in the nuclei of neurons, oligodendrocytes, and astrocytes was upregulated after brain injury, indicating that it is functionally linked to stress responses (Ariyannur et al., 2010a; Moffett et al., 2013). ACSS2 staining in the hippocampus of uninjured and injured rats shows the strong upregulation of expression in the nuclei of neurons after brain injury (Figure 7). In neuronal cell nuclei of uninjured rats, ACSS2 immunoreactivity in the majority of neurons was low and granular in appearance, possibly indicating localized clustering of ACSS2-associated transcription factor complexes at active transcription sites (Figures 7A,C). Following injury, staining became much more intense throughout neuronal nuclei indicating that upregulation of ACSS2 is required for neuronal responses to brain injury (Figures 7B,D). This increased requirement for ACSS2 in the brain’s response to injury is mostly likely associated with recycling of acetate in the cell nucleus, which is necessary when gene transcription activity is strongly elevated. However, we propose that ACSS2 upregulation in the brain is also required to make use of the concentrated brain metabolite, N-acetylaspartate (NAA), the most concentrated acetylated metabolite in the brain [reviewed in Moffett et al. (2007)]. In this view, one function of the high concentration of NAA in the brain is to act as an acetate reservoir that can be called on in response to brain injury (Moffett et al., 2013). It is noteworthy that ACSS2 was also found to be colocalized with the enzyme aspartoacylase (ASPA; discussed in more detail in section “Local Acetate Production From Acetylated Metabolites”) in the nuclei of oligodendroglioma-derived cells (Long et al., 2013). ASPA is the only known enzyme that can metabolize NAA into acetate and aspartate (Kaul et al., 1991; Namboodiri et al., 2000). We have also shown that the transport of ASPA to the cell nucleus occurs by an active process, rather than by passive diffusion (Hershfield et al., 2006). This colocalization of ACSS2 and ASPA in cell nuclei is notable because NAA can be used to support acetylation reactions in the cell nucleus in response to brain injury. The importance of NAA as an acetate source will be discussed in more detail in Section “Local Acetate Production From Acetylated Metabolites” below, as well as in part 2 of this review.

The mechanism whereby ACSS2 is translocated to the cell nucleus has been elucidated. In human glioblastoma cells, Li et al. showed that glucose limitation results in AMPK-mediated phosphorylation of ACSS2 at serine residue S659, which unmasks a nuclear localization signal sequence for importin a5 binding and nuclear translocation. Nuclear localized ACSS2 then binds to transcription factor EB and engages autophagy gene promoter regions, where ACSS2 incorporates acetate generated by histone deacetylases to generate acetyl-CoA for acetyltransferase-mediated histone H3 acetylation in these regions, thus promoting lysosomal biogenesis and autophagy (Li et al., 2017b). Other studies demonstrating the nuclear localization of ACSS2 have suggested that a significant role for ACSS2 is the generation of acetyl-CoA within the nucleus for use in histone and transcription factor acetylation reactions. Because the local supply of free acetate in the nuclei of cells is generated via the action of protein deacetylases including histone deacetylases (HDACs), it seems likely that a major role for ACSS2 is the recycling of acetate produced through all deacetylation reactions, including those mediated by sirtuins, and this has recently been demonstrated in hypoxic cancer cells (Bulusu et al., 2017). These investigators found that hypoxia increased the nuclear localization of ACSS2 and that the nuclear enzyme recaptured acetate derived from histone deacetylation reactions. The conversion of acetate to acetyl-CoA by ACSS2 is an ATP requiring reaction (Figure 5), but one that would provide efficiency in the local production of acetyl-CoA in the cell nucleus.

A systematic evaluation of histone marks published in 2011 found that 130 unique sites on histones harbored post-translational marks including lysine monomethylation, lysine dimethylation, lysine acetylation, lysine formylation and previously unknown lysine crotonylation sites (Tan et al., 2011). Crotonate is a 4 carbon unsaturated short-chain fatty acid and as such is the unsaturated (dehydrogenated) form of butyrate. Subsequently, several research groups have reported that ACSS2 may be able to utilize substrates other than acetate and that it may be involved in crotonylation of histones by generating crotonyl-CoA (Sabari et al., 2015; Jiang et al., 2018). However, Sabari and colleagues measured crotonyl-CoA concentration in HeLa S3 cells and found it to be approximately 1000-fold lower than the level of acetyl-CoA, suggesting that the contribution to histone acylation should be relatively minor when acetyl-CoA levels are plentiful. The primary evidence that ACSS2 participates in crotonylation came from knockdown of ACSS2 in cultured cells, which resulted in reduced crotonyl-CoA levels (Sabari et al., 2015). The initial report on the presence of crotonylation sites on histones outlined reasons why enzyme systems other than ACSS2 are likely involved in generating crotonyl-CoA for use in histone crotonylation (Tan et al., 2011). For example, butyryl-CoA can be converted to crotonyl-CoA by short-chain acyl-CoA dehydrogenase and glutaryl-CoA can be converted to crotonyl-CoA by glutaryl-CoA dehydrogenase. As noted above, studies on enzyme activities against various short-chain fatty acid substrates indicate that ACSS2 has a strong preference for acetate as substrate (see Table 1) making it unlikely that ACSS2 has substantial capacity to utilize crotonate as a substrate due to its larger size. However, additional studies on the full range of utilizable substrates for ACSS2 are needed.

ACSS2 has two functional roles which vary in importance based on nutritional status as well as during stress responses. In the fed state and without injury or stress, ACSS2 acts predominantly as a cytoplasmic lipogenic enzyme to facilitate lipid synthesis and storage. However, with nutrient deprivation, or during stress responses or injury, the function of ACSS2 shifts in favor of acting as a transcription factor coactivator that translocates to the nucleus along with a number of transcription factor complexes. ACSS2 is extensively involved in local acetate recycling and modulation of the metabolic state, whereas ACSS1 is more involved in the utilization of acetate for mitochondrial energy derivation. Studies on SREBP induction of ACSS2 focus on its lipogenic role, and accordingly that is what these studies highlight. However, the regulatory roles that ACSS2 plays during stress responses, hypoxia and injury would not manifest in such studies and therefore could be overlooked. Which functional role ACSS2 plays in any given physiological context will also be dependent on cell type.

### Local Acetate Production From Acetylated Proteins

There are many local sources of free acetate in all cells afforded by protein and metabolite deacetylation reactions. Protein deacetylase enzymes provide local free acetate that can be recycled by conversion to acetyl-CoA by either ACSS2 or ACSS1. Protein acetylation is one of the most ubiquitous post-translational modifications in all organisms, and deacetylation reactions provide a local supply of acetate in all subcellular compartments. Focus on this class of post-translational modification has increased in recent years as it has become apparent that it is a much more important regulatory mechanism than previously thought (Yang and Seto, 2007; Drazic et al., 2016). Acetylation and deacetylation are highly dynamic processes and the overall acetylation status of proteins in an organism is constantly shifting depending on factors such as nutritional status (Yang et al., 2011; Hebert et al., 2013; Hosp et al., 2016), exercise (Philp et al., 2014), injury (Ntranos and Casaccia, 2016), or cancer (Gong et al., 2016). The totality of this acetylation status has become known as the acetylome (Norris et al., 2009; Spange et al., 2009). Two types of protein acetylation can be distinguished, N^α^-terminal protein acetylation, which involves various amino acids at the N-termini of proteins (Polevoda and Sherman, 2003), and lysine N^ε^-acetylation, which can occur at any exposed lysine residue in a protein (Choudhary et al., 2009).

Lysine residues found throughout proteins can be acetylated and deacetylated at their ε-amino group (Yang and Seto, 2008; Zhang et al., 2009; Pehar and Puglielli, 2013). Protein acetylation and deacetylation at lysine epsilon amino groups was first studied in nuclear histones where the acetylating and deacetylating enzymes were termed histone acetyltransferases (HAT) and histone deacetylases (HDAC), respectively. However, it has become apparent that HATs and HDACs operate in the cytoplasm as well as the nucleus of cells, and that many proteins in all cellular compartments are functionally regulated by acetylation and deacetylation on exposed lysine residues (Glozak et al., 2005; Choudhary et al., 2014). For this reason, HDACs and HATs are often referred to in the literature as KDACs and KATs because the amino acid symbol for lysine is K (Yang and Seto, 2008). HDACs act to deacetylate many cytoplasmic proteins, for example, HDAC 5 acts to deacetylate tubulin in response to axonal injury (Cho and Cavalli, 2012). Many ER transiting and resident proteins are acetylated by acetyl-CoA:lysine acetyltransferases (Pehar et al., 2012b; Pehar and Puglielli, 2013). Many mitochondrial proteins are also acetylated on lysine residues, and deacetylated as part of the functional regulation of mitochondrial activity (Anderson and Hirschey, 2012). These findings indicate that lysine acetylation and deacetylation reactions occur in all major subcellular compartments including the nucleus, cytoplasm, ER and mitochondria. As such, deacetylation reactions provide local substrate for the resynthesis of acetyl-CoA via ACSS2 (nucleus, ER and cytoplasm) and ACSS1 (mitochondria).

Nutritional status has a major impact on mitochondrial protein acetylation [reviewed in Hosp et al. (2016)]. Acetylation of mitochondrial enzymes acts predominantly to inhibit enzyme activity and therefore reduces oxidative metabolism [reviewed in Baeza et al. (2016)]. High acetyl-CoA levels in the cytoplasm and nucleus may provide pro-growth conditions, whereas levels of acetyl-CoA in mitochondria that are in excess of requirements provide a signal to slow flux through oxidative energy production (Baeza et al., 2016). In recent years, evidence has been mounting for non-enzymatic acetylation of specific lysine residues in mitochondrial proteins based on the reaction conditions present in the mitochondria matrix (e.g., high pH) and the paucity of known lysine acetyltransferases in mitochondria. This has led some investigators to conclude that protein acetylation in mitochondria may be driven by mass action and is therefore predominantly non-enzymatic (Baeza et al., 2015; Davies et al., 2016). If proven true, this would make deacetylase activity (e.g., SIRT3) and removal of excess acetate and acetyl-CoA from the mitochondrial matrix critical for proper mitochondrial function. Interestingly, ACAT1, the rate limiting enzyme in ketone body formation that generates acetoacetyl-CoA, has been found to acetylate the pyruvate dehydrogenase complex in mitochondria (Fan et al., 2014). Additionally, GCN5L1 (general control of amino acid synthesis 5-like 1) has been shown to acetylate mitochondrial proteins (Scott et al., 2012). Knockdown of this acetyltransferase-like enzyme reduced mitochondrial protein acetylation indicating that some mitochondrial protein acetylation is mediated enzymatically. Activity of the mitochondrial carnitine/acylcarnitine transporter has been found to be reduced by acetylation at several lysine residues on the internal (matrix) side of the inner mitochondrial membrane, and deacetylation by SIRT3 increased activity (Giangregorio et al., 2017). It is likely that both non-enzymatic and enzymatic protein acetylation occurs in mitochondria, but it remains to be determined which mechanism is the predominant one. In other subcellular compartments, lower acetyl-CoA levels and lower pH may provide conditions less suitable for non-enzymatic lysine acetylation of proteins. In overview, mitochondria are the site of acetyl-CoA formation from nutrients, generating high levels of intramitochondrial acetyl-CoA and resulting in extensive acetylation of mitochondrial proteins. This hyper-acetylation shifts mitochondrial function away from oxidative metabolism. As such, acetyl-CoA, acetate and protein acetylation act as a nutrient sensing system that balances fuel oxidation, fuel storage and central metabolism to meet the current needs of the organism depending on nutrient availability.

The other major type of protein acetylation, N-terminal acetylation, is a prevalent cotranslational modification in eukaryotes that is mediated by N-terminal acetyltransferase (NAT) enzymes (Polevoda and Sherman, 2000; Van Damme et al., 2012). N-terminal acetylation occurs before translation is complete on the ribosome (Giglione et al., 2015). In humans, the known NAT enzymes are designated NatA through NatF. A majority of eukaryotic cytosolic proteins (estimated at approximately 80%) are N-terminal acetylated [reviewed in Aksnes et al. (2016)]. N-terminal protein acetylation serves a number of functions including regulating or facilitating protein-protein interactions. For example, acetylation of tropomyosin is required for binding to actin (Urbancikova and Hitchcock-DeGregori, 1994). Human NatB is required for maintaining the proper structure and function of actomyosin fibers and normal cellular migration, and depletion of NatB decreased acetylation of unprocessed β-actin (Van Damme et al., 2012). When N-terminal acetylated proteins are degraded through either lysosomal or proteasomal pathways, the resultant N^α^-acetylated amino acids must be acted on by aminoacylase enzymes (discussed below) in order to be further metabolized. This provides another source of local free acetate in cells.

### Local Acetate Production From Acetylated Metabolites

Acetylated metabolites are present in all tissues and some of them are detectable in human urine (Engelke et al., 2004). These metabolites can provide local sources of free acetate when appropriate deacetylase enzymes are present. Acetyl-L-carnitine (ALC) is involved in shuttling acetate groups across the mitochondrial membrane and can act as a local, intracellular source of acetate. Long-chain acyl-carnitines are transported into mitochondria to provide intramitochondrial fatty acids for β-oxidation. The acyl-carnitines are converted to acyl-CoA plus L-carnitine, and the acyl-CoA moieties can then undergo β-oxidation. L-carnitine can be combined with acetyl-CoA by the reversible enzyme carnitine acetyltransferase (CrAT, EC 2.3.1.7) to form ALC, which can be transported to the cytoplasm when mitochondrial acetyl-CoA levels are high (Figure 3). ALC is stored in the cytoplasm and is taken back up by mitochondria when needed, and reconverted into L-carnitine and acetyl-CoA by CrAT. As such, ALC acts to buffer acetyl-CoA levels and match them to metabolic requirements (Schroeder et al., 2012). CrAT is localized in the mitochondrial matrix, peroxisome core and ER lumen, but is not expressed in the cytosol (Ramsay and Zammit, 2004). As such, CrAT and ALC act to buffer the mitochondrial and ER acetyl-CoA pools.

Misfolded or non-functional proteins are recycled via lysosomal or proteasomal degradation. Proteolysis generates amino acids that are acetylated at their N-terminus and these must be deacetylated before they can be further metabolized. Deacetylation of N^α^-acetylated amino acids is accomplished by enzymes known as aminoacylases. Three aminoacylase enzymes are expressed in mammals. Aminoacylase-1 is the enzyme responsible for the deacetylation of most N^α^-acetylated amino acids with the highest activity against acetylated methionine, leucine, glutamate, and glutamine (Lindner et al., 2008). Aminoacylase-3 deacetylates N-acetyl-aromatic amino acids (Pushkin et al., 2004; Tsirulnikov et al., 2009). In contrast, aminoacylase-2, which is also known as aspartoacylase (ASPA), acts on only one known substrate; N-acetylaspartate or NAA. Unlike N^α^-acetylated amino acids associated with the N-terminus of proteins, NAA is synthesized by a unique enzyme, aspartate N-acetyltransferase (Asp-NAT; gene *NAT8L*) which is expressed very strongly in the nervous system (Truckenmiller et al., 1985; Madhavarao et al., 2003). NAT8L is also expressed in adipose tissue (Pessentheiner et al., 2013; Prokesch et al., 2016). NAA is present in the human brain at concentrations exceeding 10 mM, rivaling glutamate as the most concentrated brain metabolite [reviewed in Moffett et al. (2007)]. In the nervous system, NAA is synthesized in neurons from aspartate and acetyl-CoA and supplies approximately 1/3 of the carbon required for myelin lipid synthesis during brain development (Madhavarao et al., 2005; Wang et al., 2009). NAA is highly concentrated in neurons (Moffett et al., 1991; Moffett and Namboodiri, 1995, 2006) and is thought to be involved in acetyl-CoA dependent functions in the nervous system (D’Adamo et al., 1968; Choi and Gruetter, 2004; Ariyannur et al., 2010b; Moffett et al., 2013). In order for NAA to be metabolized, it must first be deacetylated by ASPA. However, ASPA is mostly expressed in oligodendrocytes (Klugmann et al., 2003; Madhavarao et al., 2004; Moffett et al., 2011), the myelin forming cells of the central nervous system. This requires that NAA synthesized in neurons be transferred to oligodendrocytes for catabolism and release of free acetate. Oligodendrocytes, in turn, express ACSS2 in their nucleus and cytoplasm, and therefore can convert the free acetate into acetyl-CoA for use in myelin lipid synthesis and other acetyl-CoA related functions (Ariyannur et al., 2010a). As such, NAA represents one of the most concentrated sources of acetate in the human body, and is likely to supply oligodendrocytes with a proportion of the substrate for acetyl-CoA synthesis, especially during brain development and myelination. The role of NAA in acetate metabolism will be discussed in more detail in part 2 of this review.

To summarize, local free acetate can be produced by (1) deacetylation of acetylated proteins as part of normal regulatory processes, (2) deacetylation of amino acids that result from the proteolysis of acetylated proteins in lysosomes, proteasomes, etc., and (3) deacetylation of enzymatically synthesized metabolites such as acetyl-CoA, acetyl-carnitine and NAA. This local production provides for intracellular carbon transport and recycling, which is more critical at times of starvation when carbon sources in the diet are absent. The kidney expresses all 3 aminoacylase enzymes involved in amino acid and metabolite deacetylation and acts as a final reclamation point for acetate derived from acetylated molecules.

## Similarities and Differences Between Ketone Bodies and Acetate

**FIGURE 8:**
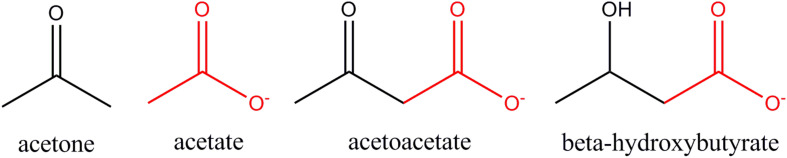
Structural similarities between acetate and ketone bodies. Three are monocarboxylic acids, including acetate, whereas acetone does not contain a carboxyl group (COO^–^). Acetate is structurally identical to the carboxyl terminus of both acetoacetate and β-hydroxybutyrate (red highlight).

Ketone bodies and acetate are structurally similar (Figure 8). With the exception of acetone, all are monocarboxylic acids with a net negative charge at physiological pH. Despite these close similarities, the enzymes involved in the metabolism of acetate and ketone bodies are distinct (Table 2), and this may be one of the reasons why acetate has not been emphasized as playing a significant role in carbon mobilization under ketogenic conditions in humans. The utilization of ketone bodies in target tissues requires an enzyme that is not present in the liver’s ketone body metabolic pathway, β-ketoacyl-CoA transferase, which converts the ketone body acetoacetate to acetoacetyl-CoA. The lack of this enzyme in hepatocytes limits their capacity to utilize ketone bodies. In contrast, ACSS2 is strongly expressed in the liver indicating that acetate produced in the liver during fasting can be reconverted to acetyl-CoA.

**TABLE 2:** Differences in the metabolism of ketone bodies and acetate.

***Ketone bodies***	***Acetate***
**Enzymes involved in synthesis**	
(1) Acetyl-CoA C-acetyltransferase	(1) Acyl-CoA thioesterase 12 (acetyl-CoA hydrolase)
(2) Hydroxymethylglutaryl-CoA synthase	(2) Histone and protein deacetylases (HDAC/KDAC)
(3) Hydroxymethylglutaryl-CoA lyase	(3) Sirtuins
(4) 3-hydroxybutyrate dehydrogenase	(4) Metabolite deacetylases (e.g., aminoacylases, etc.)
**Enzymes involved in utilization**	
(1) 3-hydroxybutyrate dehydrogenase	(1) acyl-CoA short chain synthetase-1 (cytoplasm/nucleus)
(2) β-ketoacyl-CoA transferase	(2) acyl-CoA short chain synthetase-2 (mitochondria)
(3) Acetyl-CoA C-acetyltransferase	
**Organs that release systemic ketone bodies and acetate**	
Liver, Kidney	Liver, Intestine, Kidney and others

*Even though circulating ketone bodies and systemic acetate serve the same function of redistributing carbon sources, they are generated and metabolized by different enzyme systems.*

Acetate and ketone bodies are the final products of fatty acid β-oxidation in the liver during fasting conditions, and the bulk of these metabolites are not utilized in the liver. Instead, they are released to the circulation to be metabolized in extra-hepatic tissues. The end product of ketone body and acetate metabolism in extra-hepatic tissues is acetyl-CoA. As such, ketone bodies and acetate represent mechanisms for the redistribution of acetyl-CoA from the liver to other tissues when externally supplied nutrients are not available. In humans, acetate is thought to be produced primarily through the action of ACOT12, which is localized in the cytoplasm of hepatocytes. In mammals such as rodents, ACOT12 is localized in both the cytoplasm and peroxisomes, increasing the capacity of hepatocytes to produce free acetate. It is possible that ACOT8, which is localized to peroxisomes in humans, produces some acetate, but this remains to be determined (Hunt et al., 2014). Ketone bodies, in contrast, are synthesized predominantly in the mitochondria of hepatocytes. Ketogenesis has also been found to occur to a lesser extent in the kidneys (Nakatani et al., 1996, 1999; Takagi et al., 2016). Despite the fact that acetate and ketone bodies are synthesized to some extent in different subcellular compartments, and are produced and metabolized by different enzyme systems, they can serve virtually identical functions in redistributing key sources for acetyl-CoA synthesis in extra-hepatic tissues.

One additional similarity between acetate and ketone bodies is how they are transported across cell membranes. Ketone bodies and acetate must be transported bidirectionally to be released from hepatocytes and taken up by target cells throughout the body. Transmembrane transport is mediated by four members of the SLC16 gene family; the monocarboxylate transporters MCT1 – 4. These transporters mediate proton-linked transport of monocarboxylates including pyruvate, lactate, ketone bodies (Halestrap, 2013) and acetate (Moschen et al., 2012). Ketone bodies and acetate are transported predominantly by MCT1, MCT2 and to a lesser extent by MCT4, whereas MCT3 is involved in lactate transport. There is also evidence that acetate may be passively transported into or out of some cells through aquaporins (Ferro et al., 2016).

The production of classical ketone bodies and acetate from the same source, acetyl-CoA, in the liver during starvation begs the question of why distinct substrates are synthesized and metabolized by distinct routes. A possible explanation is that ketone bodies and acetate are targeted to somewhat different organ systems under ketogenic conditions. Studies on *ACSS1* knockout mice provide some clues along these lines. As noted above, ACSS1 is the mitochondrial form of the enzyme and it is highly expressed in heart, skeletal muscle, and brown adipose tissue. *ACSS1* knockout mice appear normal when well fed, but upon fasting they develop hypothermia due to lack of acetate conversion to acetyl-CoA in brown adipose tissue. When fasted, these mice also have reduced exercise capacity and muscle ATP levels because acetate cannot be oxidized for energy (Sakakibara et al., 2009). These findings suggest that acetate may be most critical for muscle and heart activity, as well as thermogenesis, during starvation. In contrast, ketone bodies are more broadly targeted to almost all tissues of the body during starvation, including the brain.

Another difference between ketogenesis and acetogenesis is that acetate production is less centralized, and not dependent upon hepatocytes exclusively. Acetate is produced and released in other tissues, including the intestine where much of the acetate is of bacterial origin. Acetate is also produced locally in all cells during the normal course of deacetylation reactions of all types. Therefore, ketogenesis primarily involves acetyl-CoA produced via β-oxidation of fatty acids, whereas acetogenesis involves other sources including hydrolysis of existing acetyl-CoA and deacetylation of acetylated metabolites and proteins.

One final common aspect of the functional roles that acetate and ketone bodies play is that they both take part in metabolite sensing and epigenetics through post-translational modifications of proteins involved in the regulation of metabolism and gene transcription. In this role these metabolites are used to modify lysine residues on the surface of receptors, enzymes and transcription factors involved in the regulation of central metabolism as well as histones involved in chromatin remodeling for gene transcription (reviewed in Zhao et al., 2018).

## Acetate, ACSS2 and Metabolic Regulation Through AMPK

The ATP-requiring enzymatic conversion of acetate and CoA to acetyl-CoA and AMP is an evolutionarily ancient and conserved metabolic pathway (Karan et al., 2001; Ingram-Smith and Smith, 2007). This metabolic pathway provides a sensitive measure of cellular energy charge and can be useful for fine tuning anabolic and catabolic processes. As such, it is not surprising that AMPK (adenosine 5′-monophosphate-activated protein kinase) has been found to regulate ACSS2 function and subcellular location.

AMPK is often described as a master regulatory enzyme that controls central energy metabolism (Winder and Hardie, 1999). AMPK is a serine/threonine kinase that has a major role in energy metabolite sensing and responses to shifting energetic requirements (reviewed in Kahn et al., 2005; Wang and Lei, 2018). AMPK responds to decreasing cellular energy status by stimulating catabolism and energy derivation while also inhibiting biosynthetic pathways in order to replenish cellular energy supplies [reviewed in Hardie et al. (2012)]. As such, AMPK activity is regulated by nutritional status and the degree of energy expenditure (Hardie, 2011). In this role, AMPK acts to phosphorylate and regulate key enzymes involved in a number of major metabolic pathways. The overall effect of AMPK activation and the subsequent phosphorylation of its protein targets is to induce glycolysis, lipolysis, hepatic ketogenesis, fatty acid β-oxidation and autophagy. Simultaneously, anabolic pathways such as lipid and sterol synthesis and gluconeogenesis are inhibited. AMPK is also involved in the regulation of food intake based on its activity in the hypothalamus, which is responsive to nutritional and hormonal signals (Minokoshi et al., 2004; Nicolaidis, 2011).

**FIGURE 9:**
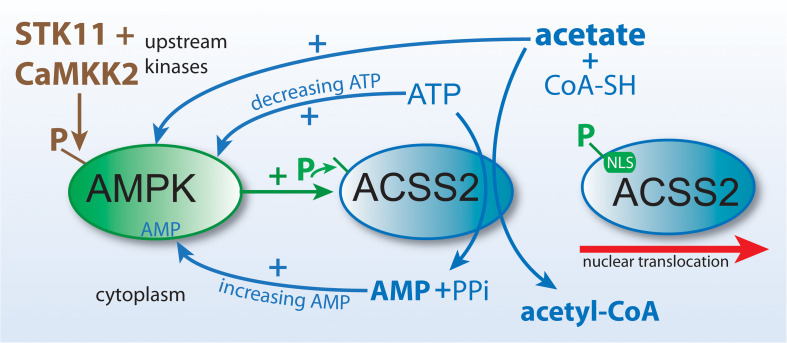
Hypothetical role of AMPK in ACSS2 task switching and translocation. Nutrient depletion induces a kinase cascade in which AMPK is activated by upstream kinases and in turn acts to phosphorylate downstream target proteins to enhance energy derivation and limit non-essential energy requiring activities. Lipogenesis is inhibited, but paradoxically ACSS2, which is thought to be a lipogenic enzyme, is activated by AMPK-mediated phosphorylation (P). The enzymatic action of ACSS2 drives ATP levels down and AMP levels up, both of which act to increase AMPK activity, forming a positive feedback loop. Acetate may also activate AMPK. Phosphorylation of ACSS2 exposes a nuclear localization signal sequence (NLS), leading to the translocation of ACSS2 to the cell nucleus. This shifts ACSS2 from a cytoplasmic lipogenic function to a nuclear regulatory role where acetyl-CoA formation becomes involved in histone and transcription factor acetylation.

Multiple mechanisms are involved in the activation of AMPK in response to nutrient deprivation and energy expenditure (Winder and Hardie, 1999; Zong et al., 2019). These include activation via phosphorylation by upstream kinases including serine/threonine kinase 11 (STK11, also known as LKB1) and calcium/calmodulin-dependent protein kinase kinase 2 (CaMKK2) and allosteric activation by low ATP levels and increased levels of AMP and ADP (Xiao et al., 2011). AMPK is also activated indirectly by low glucose levels (Lin and Hardie, 2018). The most critical mechanism involves activation of AMPK by upstream kinases that target threonine 172 for phosphorylation (Figure 9), which is required for increasing AMPK activity (Willows et al., 2017). AMPK is also allosterically activated by increased intracellular concentrations of AMP (Moore et al., 1991; Gowans et al., 2013). The intracellular AMP concentration is typically 10-fold lower than that of ATP, making AMP a sensitive measure of the cellular energy charge (Sanders et al., 2007). How AMPK specifically responds to various stimuli depends on which isoforms of the regulatory subunits constitute the heterodimeric complex (Ross et al., 2016).

This brings us to the question of the role of acetate in the regulation of AMPK and metabolism. Acetate administration has been shown to increase AMPK phosphorylation and activation. Initial studies were done *in vivo* in rats that were fed a diet including 0.3% acetate. In these studies, dietary acetate led to reduced expression of genes involved in gluconeogenesis and lipogenesis in the liver and directly activated AMPK (Sakakibara et al., 2006). Subsequent *in vitro* studies using bovine hepatocytes indicated that acetate application led to decreased cellular ATP and a concomitant rise in AMP and the AMP/ATP ratio (Li et al., 2013). This was accompanied by a dose-dependent increase in AMPK phosphorylation and AMPK activity. These investigators found that acetate activation of the AMPKα signaling pathway led to increased lipid oxidation and decreased lipid synthesis. Later studies using a rat liver cell line confirmed that acetate administration increased AMPKα phosphorylation (Li et al., 2018). These findings make physiological sense if systemic acetate, like ketone bodies, signals mobilization of energy reserves where lipolysis and fatty acid β-oxidation are central. However, if ACSS2 is a lipogenic enzyme then it would be expected that AMPK would antagonize its actions. AMPK-mediated phosphorylation regulates lipid synthesis and lipid oxidation by activating fatty acid oxidation enzymes, inhibiting fatty acid synthesizing enzymes, or by altering the subcellular localization of target proteins (Wang et al., 2018).

As discussed earlier, in order for acetate to be metabolized for energy derivation or lipid synthesis, it must be converted to acetyl-CoA either by ACSS1 in mitochondria or ACSS2 in the cytoplasm or nucleus. Both enzymes utilize ATP and generate AMP and therefore would tend to activate the AMPK pathway to replenish the cellular energy charge. The acetate activated intra-mitochondrially by ACSS1 can be readily oxidized to CO_2_ for energy derivation, but acetate metabolized by ACSS2 has been thought of as being destined for lipid synthesis. AMPK antagonizes lipogenesis, and as ACSS2 is a known lipogenic enzyme, it would be expected that AMPK would inhibit ACSS2 action. Recent findings clarify the relationship between ACSS2 and AMPK and provide connections to autophagy. Li et al. have reported that phosphorylation of ACSS2 at serine 659 by AMPK unmasks a nuclear localization sequence on ACSS2 and induces nuclear translocation (Li et al., 2017a). This may help solve the paradox that ACSS2 is the target of a kinase that is involved in increasing lipid oxidation and reducing lipid synthesis. Based on the available evidence, we propose that there is bidirectional signaling between ACSS2 and AMPK that may form a positive feedback loop in certain physiological circumstances, for example very high acetate concentrations. However, during fasting or cellular stress reactions (e.g., hypoxia) AMPK diverts ACSS2 from a lipogenic to a regulatory function. The ACSS2 enzymatic reaction generates AMP, which in turn acts as a signal for declining cellular energy reserves. By swapping acetate and ATP for acetyl-CoA and AMP, ACSS2 acts to drive both protein acetylation and AMPK activation. AMPK phosphorylation of ACSS2 increases acetyl-CoA formation while also decreasing ATP and increasing AMP. These in turn produce feedback to increase AMPK activity allosterically (Figure 9). Li and colleagues found that nuclear localized ACSS2 forms a complex with transcription factor EB (TFEB) and utilizes the acetate generated from histone deacetylation to locally produce acetyl-CoA for additional histone acetylation in the promoter regions of TFEB target genes. TFEB is a critical transcription factor involved in lysosomal biogenesis and autophagy.

We propose that ACSS2 is directly involved in metabolic reprogramming associated with nutrient and oxygen deprivation, as well as other critical cellular functions in many different cell types. Whereas ACLY provides the bulk of acetyl-CoA in all subcellular compartments, ACSS2 is unique in that it can task switch from a cytoplasmic to a nuclear role on demand. During cellular stress responses, AMPK phosphorylates ACSS2 as AMP levels rise, and this, in turn, unmasks the nuclear localization sequence and results in translocation to the nucleus where ACSS2 associates with open chromatin and a number of transcription factors. In order to reprogram metabolism, ACSS2 utilizes the acetate derived from histone and other nuclear protein deacetylation reactions to form acetyl-CoA within transcription factor complexes and in turn acetylate and modulate their activity. This role in metabolic reprogramming is particularly important for the discussion on acetate and cancer in Part 2 of this review.

## Conclusion Part 1

ACSS2 function clearly has survival and reproductive value as it is well conserved phylogenetically. Loss of ACSS2, however, is not embryonic lethal and causes no overt pathology in well fed adult mice. This suggests that ACSS2’s actions are modulatory, acting to adjust various signaling pathways, rather that turning them on or off directly. ACSS2 function is both tissue specific and compartment specific within cells. Cytoplasmic ACSS2 is involved in lipid synthesis and protein acetylation reactions, including cytoskeletal and ER protein acetylation reactions. The nuclear translocation of ACSS2 no doubt acts to shift its function away from lipogenesis and other cytoplasmic roles to the regulation of gene expression. This occurs during nutrient limitation and other stressors including injury, hypoxia and inflammation. Locally produced acetate has many specialized functions including acting as a nutrient deprivation and stress-indicator, for example during hypoxia, as well as providing substrate for protein and metabolite acetylation reactions that control protein function. ACSS2 can reclaim locally produced acetate in the cytoplasm, ER lumen, and nucleus, increasing efficiency over reliance on mitochondrial-derived citrate. Specificity in the epigenetic regulation of cell function can be imparted by ACSS2 recruitment to and activation of transcription factor complexes through acetylation mediated by proteins such as CREB binding protein (CBP) or similar coactivators (e.g., p300). By directly providing the acetyl-CoA needed for transcription factor acetylation, ACSS2 may act as a signal amplifier or dampener in transcriptional regulation, depending upon the transcription factor complex involved. ACSS2 further shapes the epigenetic landscape through alterations in histone modification. Histone acetylation tends to increase access of transcriptional complexes to specific DNA sites, whereas histone methylation tends to repress access and gene expression. Cross-talk between histone acetylation and methylation systems acts to coordinate control over chromatin remodeling and transcriptional access to DNA (Ye and Tu, 2018), and ACSS2 may play a role in this coordinated activity. Many questions remain concerning the sources of free acetate in normal and cancerous cells under different physiological conditions, and the significance of acetate release from various sources vs. acetate uptake and utilization. In part 2 of this review, we will focus on the roles of acetate and ACSS2 in various disease states, including cancer.

## Author Contributions

JM and AN conceived the project. All authors wrote the manuscript.

## Conflict of Interest

The authors declare that the research was conducted in the absence of any commercial or financial relationships that could be construed as a potential conflict of interest.

## Footnotes

1 https://www.ncbi.nlm.nih.gov/biosample/
